# The feasibility and efficacy of coach-led virtual home-based cycling among individuals with cerebral palsy

**DOI:** 10.3389/fneur.2025.1604061

**Published:** 2025-07-15

**Authors:** Lisbeth Hoejkjaer Larsen, Henrik Kirk, Jakob Lorentzen

**Affiliations:** ^1^Department of Neuroscience, University of Copenhagen, Copenhagen, Denmark; ^2^Department of Paediatrics, Copenhagen University Hospital Rigshospitalet, Copenhagen, Denmark; ^3^Neuropotentiale, Physiotherapy Clinic, Copenhagen, Denmark

**Keywords:** home-based, virtual cycling, cerebral palsy, feasibility, high-intensity exercise

## Abstract

**Introduction:**

Cerebral palsy (CP) is a neurological disorder that impairs motor control and coordination, often leading to physical and social restrictions in daily activities. This single-arm feasibility study investigates the potential efficacy of virtual moderate- to high-intensity cycling at home among individuals with CP.

**Methods:**

Twenty-three individuals with CP (*GMFCS I-IV; 16 males; mean age 26, range 13–58 years*) were recruited for a 12-week home-based cycling intervention. The intervention included three weekly sessions, which could be completed either in an online coach-led group setting or independently, along with guidance for additional training. The primary focus was feasibility, addressed by retention, adherence, and safety. Efficacy was primarily evaluated using a functional threshold power test for cycling (FTP), the Timed Up and Go Test (TUG), and the Sit to Stand test (STS), assessed before and after the intervention, and secondly via self-reported questionnaires.

**Results:**

The intervention was demonstrated to be safe and feasible, with no adverse events reported. Retention was high, with only one dropout attributed to mononucleosis. The remaining 22 participants completed the study with a high attendance averaging 3.1 sessions/week [range 2–5]. Improvements were observed in the FTP test (67.2 ± 37.3 W, *p* < 0.001), the TUG test (2.1 ± 1.4 s, *p* < 0.001), and the STS test (3.9 ± 3.3 repetitions, *p* < 0.001) while self-reported fatigue, pain, sleep, well-being, and self-efficacy remained unchanged.

**Discussion:**

Our findings suggest that virtual cycling at home is a safe and feasible approach to engage in moderate- to high-intensity exercise, enhance physical capacity, and improve functional activity outcomes for individuals with activity limitations.

## Introduction

Individuals with cerebral palsy (CP) have a higher rate of chronic conditions and multimorbidity compared to the general population (1). Studies demonstrate that individuals with CP experience significant progressive functional declines, fatigue, diminished musculoskeletal mass and quality, excess adiposity, chronic physical inactivity, and increased risk for cardiovascular disease all of which increase with age (2, 3). Studies have also identified that aging with CP is accompanied by higher rates of chronic pain (4) and sleep problems (2). These factors contribute to an observed increase in the prevalence of mental health issues and a decline in quality of life among individuals with CP compared to the general population (5, 6). Preventive strategies to address both physical and mental health concerns are therefore highly needed. However, participation in physical and social activities can be hindered for many individuals with CP due to mobility impairments and the need for assistive devices. Inaccessible public spaces, lack of transportation, and insufficient support services further limit their ability to engage in these activities. Moreover, individuals with CP may face discrimination or social stigma, leading to lower confidence in managing challenges, exclusion, or isolation.

In recent years, the application of virtual reality to home-based interventions has gained increasing interest, with multiple studies conducted among children with CP (7, 8). Less research has been conducted on home-based virtual reality interventions for adolescents and adults with CP. A recent study by Holmes et al. (9) showed that home-based cycling is feasible for non-ambulant adults with cerebral palsy (CP). However, the study had a short duration of only 4 weeks, involved a small sample size of just 10 participants, and did not incorporate a virtual environment. Riding together (virtual and online) while apart (at home) may enable moderate- to high-intensity training by overcoming environmental and social barriers, eliminating the need for transportation, and supporting online social interaction with peers. The intensity is ensured through the activation of large muscle groups during cycling, while the seated position allows individuals with balance impairments or reduced physical function to perform the activity safely. Technological advancements have enabled the development of more user-friendly, cost-effective, enriched, and reliable solutions for home-based training, enhancing their overall effectiveness and accessibility (10). In addition, modern health technology is often integrated with evidence-based behavior-regulating strategies, including self-monitoring, feedback, goal setting, and reward mechanisms. Moreover, integrating the home-based setting with online group sessions supervised by a coach can facilitate social engagement and interaction. Thus, with guidance on progressive challenges and variations, home-based cycling may provide a convenient solution for enhancing training volume (10).

The primary aims of this study were to evaluate the safety and feasibility, and the effects of a home-based intervention using a virtual cycling universe on cycling performance and functional activity outcome measures among individuals with CP. Secondary aims were to explore the effects on self-reported measures of fatigue, pain, sleep, well-being, and self-efficacy.

## Materials and methods

### Participants

Twenty-three individuals with CP were recruited for the study. Participants were recruited through advertisements placed in relevant clinics and an organization that support people with CP. The wide age range was selected because of the early onset of functional decline that continues to progress throughout life. To be eligible, participants had to be within the age range of 13 to 60 years and demonstrate the ability to maintain balance while riding a stationary bicycle. All participants gave informed consent to the experimental procedure, which was approved by the local ethics committee (H-22032100) and registered in ClinicalTrials.gov (NCT06402799). The study was performed in accordance with the Declaration of Helsinki.

### Procedures and outcome measures

Feasibility was a primary outcome of interest and encompassed retention, adherence, and safety. Retention was evaluated based on the proportion of participants who attended both the baseline and post-intervention assessments, compared to the total number of participants who completed the baseline assessments, with a retention target set at a minimum of 95%. Adherence was measured through participant activity logs on the training platform Zwift, with a success criterion of attending an average of 2 out of 3 weekly sessions. Safety was assessed by recording any adverse events that occurred during sessions (e.g., falls or injuries that required medical attention and/or hindered continued participation) as well as minor incidents that did not prevent participants from completing the planned training volume. The study’s success criterion was an absence of adverse events.

A physical therapist with expertise in CP assessed the GMFCS levels and types of CP. The same therapist conducted evaluations for all participants before and after the 12-week intervention, at their homes. The tests were carefully explained, and familiarization was ensured before they were administered. A Timed Up and Go Test (TUG) was performed to measure dynamic balance and mobility (11). This assessment was conducted using a standard chair without armrests, three meters of free floor space, and a stopwatch. The participant was asked to stand up, walk 3 meters to a line on the floor, and return to a seated position in the chair while being timed. A therapist was nearby to secure a safe test setting and avoid falling. In addition, the Sit to Stand test (STS) was used as a functional measure of lower body strength and endurance (12). The participant was seated in the standard chair with arms crossed (if possible) over the chest and was instructed to stand up fully and sit down as many times as possible within 30 s. To assess cycling performance, participants completed a modified Functional Threshold Power test for cycling (FTP). An indoor bicycle with toe clips (Taurus Smart Bike Z9.9 Pro, Lingen, Germany) or a home-trainer (Wahoo KICKR CORE Zwift One, Atlanta, US attached to the participants’ personal bicycles) was provided and set up in their homes. The use of an indoor bicycle or a home-trainer was determined by the participant’s functional level. Participants with the lowest functional level used an indoor bicycle, as it is easier to mount and offers greater stability due to its fixed handlebar. The FTP test involved a maximal effort ride on the stationary bicycle starting at either 50 W (Ramp Test Lite) or 100 W (regular Ramp Test) depending on the participant’s functional level. The Ramp Test Lite commenced at 50 W, with resistance increasing by 5 W per minute until the point of exhaustion. The regular Ramp Rest was initiated at 100 W, with resistance increasing by 10 W per minute until exhaustion (see Appendix 1 for further details on the modified FTP tests). When the test is completed, the FTP is calculated as 75% of the highest 1-min power averaged during the test, providing an estimate of the individual maximum power that can be maintained over longer durations (30–70 min). In addition to assessing cycling performance, this FTP test was also used to personalize resistance in the intervention workouts, as explained further in the intervention section.

Participants received questions by email before and after the training period regarding their current level of fatigue (FSS) (13), Pain (VAS scale) (14), Well-Being (WHO5) (15), Sleep problems (16), and Self-efficacy (Gses) (17). At the end of the study, participants were also asked to provide feedback on the three most positive and negative aspects of the study, respectively.

### Intervention

A virtual training platform (Zwift Inc., Long Beach, United States) was used for the intervention. This platform features a variety of virtual workout programs, along with tools for tracking performance metrics and setting personal goals. The training program was designed by a member of the research team, initially focusing on developing technical skills to prevent injuries and enabling training at intensities that produce a cardiovascular effect. The training program consisted of three sessions per week. The workouts included various forms of interval training, with effort levels carefully controlled through the Ergometer (ERG) mode in Zwift. This mode automatically adjusts cycling resistance to maintain a prescribed power output, regardless of cadence. Power output is calculated as the product of torque (pedal force) and cadence (pedal speed). In ERG mode, if cadence decreases, resistance increases accordingly, and vice versa, ensuring the target power output is consistently maintained throughout the workout. The prescribed power output was based on each participant’s FTP test, ensuring that all participants reached the intended intensity levels. The length of the scheduled training gradually increased during the intervention period from 78 min in the first week to 245 min in the last week. For more detailed information, please see Appendix 2, which includes the complete training program. The group sessions were carried out online in Zwift and combined with simultaneous Microsoft Teams (Microsoft Corporation, Redmond, US) sessions providing supervised training and allowing participants to see and hear each other. In Zwift, the sessions were arranged as “Keep everyone together” and with “Meetup-only View,” settings that ensure no one is left behind while enhancing the team cycling experience. The Microsoft Teams sessions were led by an experienced coach, who provided verbal guidance on topics such as bike positioning, cadence, sensation of pulse and fatigue, and respiration during the sessions. Furthermore, the coach suggested specific Zwift workout programs that could complement the group sessions. Participants were encouraged to attend the online group sessions, but they were allowed to complete the planned workout program in Zwift independently at an alternative time. Aside from the intervention, the participants were instructed to continue their regular activities and maintain their usual lifestyle throughout the intervention period.

### Statistics

The sample size for this study was 23 participants, which we estimated to be adequate to test the feasibility and effect of the home-based cycling intervention based on prior similar studies (18). The feasibility outcome measure was evaluated by retention, adherence, and safety. To compare physical and self-reported outcome measures before and after the intervention, data were assessed for normality using the Lilliefors test. A complete case analysis was conducted, meaning that only participants with pre- and post-data were included. Parametric comparisons were conducted using paired t-tests and corresponding 95% confidence intervals. When the t-test was used, the effect size was given as Cohen’s d and interpreted as small (0.2), medium (0.5), or large (0.8). For non-parametric data, Wilcoxon signed-rank tests were used and 95% confidence intervals for median differences estimated using bootstrap resampling. The effect size was calculated as rank-based r *(r = Z/√N)* and interpreted as small (0.1), medium (0.3) or large (0.5). Statistical significance was set at *p* ≤ 0.05. Statistical adjustments for multiple testing were conducted using Bonferroni-adjusted *p*-values for primary outcome measures. Secondary outcome measures were not included in the Bonferroni correction due to their exploratory nature.

## Results

**Figure 1:**
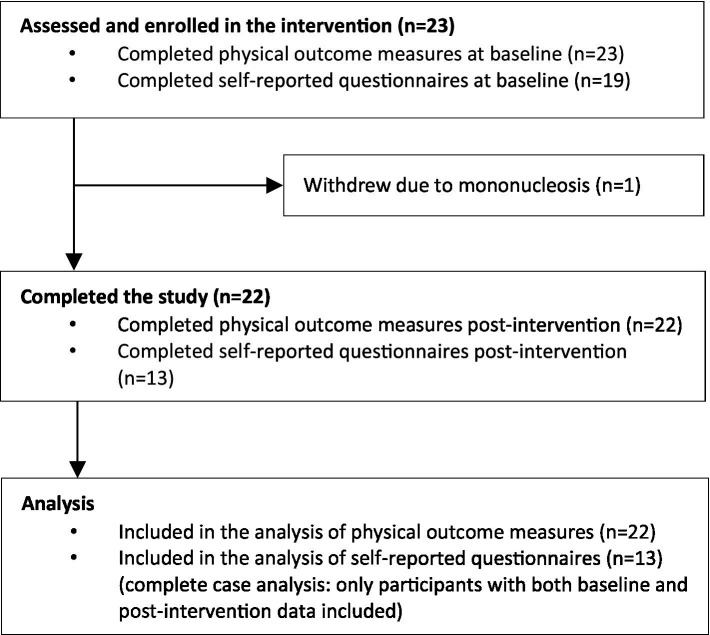
Flow diagram.

Twenty-two (96%) individuals completed the intervention. One individual had to drop out shortly after the intervention began due to mononucleosis. A flow diagram is presented in Figure 1. Demographic characteristics are shown in Table 1. Adherence was high. During the 12-week intervention period participants completed a mean of 37 sessions with a range of 24–57 sessions corresponding to a mean of 3 (range 2–5) sessions per week or 153 (range 76–414) minutes/week. No adverse events occurred that required medical attention or hindered ongoing participation. However, one participant reported experiencing leg cramps following the most high-intense workout sessions, while another complained about pressure on his prostate. In both cases, no treatment was required, and the participants were able to continue with the intervention.

**Table 1:** Characteristics of participants (*n = 22*).

Characteristic	Value
Mean age (range years)	26 (13–58)
Gender (n males/n females)	16/6
Motor ability (GMFCS)	
I (*n*)	12
II (*n*)	8
III (*n*)	1
IV (*n*)	1
Type^*^	
Hemiplegia (*n*)	8
Diplegia (*n*)	8
Triplegia/Quadriplegia (*n*)	6

^*^Hemiplegia affects movement and muscle tone on one side of the body (one arm and one leg), diplegia affects two limbs (most commonly the legs), while triplegia and quadriplegia affect three and four limbs, respectively.

Three participants conducted a ramp test lite (50 W) while 19 performed a regular ramp test with 100 W. Two participants needed arm support when transitioning from sitting to standing, while two required brief support when making the turn in the TUG test. The individual test adaptations at baseline were repeated after the intervention to ensure pairwise comparability. The mean cycling performance increased with 67.2 ± 37.3 W from baseline to after the intervention (*p* < 0.001), the time for the TUG test decreased by 2.1 ± 1.4 s (*p* < 0.001), and the repetitions in the STS test increased with 3.9 ± 3.3 (*p* < 0.001) (Table 2). No changes were observed in self-reported questionnaires on fatigue, pain, well-being, sleep issues, or self-efficacy. However, only 60% (*n* = 13) of the participants completed the online questionnaires.

**Table 2:** Explorative statistics of secondary outcome measures.

Parameter	Baseline	Post	Diff.	*p*-value	Bonf. *p*	95% CI	Effect size
FTP (Watt) *n* = 22	116.6 ± 41	183.8 ± 63.7	67.2 ± 37.7	<0.001^*^	<0.001^*^	(50.7; 83.7)	*d =* 1.8
TUG (seconds) *n* = 22	9.1 ± 4.9	7.0 ± 4	−2.1 ± 1.4	<0.001^*^	<0.001^*^	(−2.7; −1.3)	*r* = 0.9
STS (repetitions) *n* = 22	13.8 ± 5.3	17.7 ± 5.2	3.9 ± 3.3	<0.001^*^	0.002^*^	(2.0; 6.5)	*r* = 0.8
Fatigue (FSS) *n* = 13	35.7 ± 12.5	33.6 ± 14.8	−2.1 ± 5.7	0.21	-	(−5.5; 1.4)	*d = 0.4*
Pain (VAS) *n* = 13	2 ± 1.7	2.3 ± 1.4	0.2 ± 1.4	0.58	-	(−0.6; 1)	*d = 0.2*
Well-Being (WHO5) *n* = 13	61.2 ± 14.9	62.8 ± 16.7	1.5 ± 8.4	0.52	-	(−3.5; 6.6)	*d = 0.2*
Sleep (Jenkins scale) *n* = 13	6.9 ± 5.3	6.2 ± 4.7	−0.8 ± 2.4	0.27	-	(−2.2; 0.7)	*d = 0.3*
Self-efficacy (Gses) *n* = 13	29.8 ± 5.6	29.5 ± 4.9	−0.2 ± 2.6	0.75	-	(−1.8; 1.3)	*d = 0.1*

Note that values at Baseline and Post and Diff. are expressed as means ± SD values; *n* = 22 for physical tests (FTP, TUG and STS); *n* = 13 for self-reported questionnaires (Fatigue, Pain, Well-being, Sleep and Self-efficacy). Effect size is given as Cohen’s d for t-test and Rank-based r (r = Z/√N) for Wilcoxon signed-rank tests. The 95% confidence interval is based on the mean difference for the t-test and on the median difference for the Wilcoxon signed-rank test. Diff, difference; Bonf. p, Bonferroni-adjusted *p*-value; CI, confidence interval; *, statistically significant (*p* ≤ 0.05).

Not everyone prioritized the group-based sessions and completed the training programs independently. Thus, the individual participation in the group-based training sessions ranged from 1–33 sessions (out of 36 sessions) during the intervention with a mean of 19 sessions among all participants. No correlation was found between the completed training volume and participation rate in the group-based sessions (R^2^ = 0.26).

The evaluation questionnaires highlighted several positive aspects of the study, including a supportive peer community, improved physical function and capacity applicable to other activities, increased self-esteem, and an overall better mood. Most participants cited the self-monitoring aspect as a positive feature, while one participant had the opposite experience, feeling that the embedded features of quantitative measurements such as distance and pace negatively impacted motivation. Additional negative aspects mentioned in the questionnaire included technical difficulties with the setup, pain-related issues, and the scheduled training time for the group sessions.

## Discussion

This study investigated the feasibility, safety, and effect of a coach-led virtual home-based cycling intervention among individuals with CP. Results suggest that the home-based training program is feasible, and safe, and may enhance cycling performance and functional activity outcomes.

### Feasibility and compliance

Overall, the intervention was feasible. Retention and adherence were high, and no adverse events occurred. The success criterion of attending an average of 2 out of 3 weekly sessions was achieved by all participants (range 2–5 sessions/week), corresponding to a group adherence rate of 100% (on average 3 sessions per week). This is in accordance with previous studies, which reported adherence rates ranging from 75 to 96% (18). We observed considerable variability in the training volume among participants, ranging from 76 to 414 min per week, partly due to the embedded opportunity to complement the group training sessions with additional suggested workouts. Furthermore, a vast difference in physiological baseline levels and function may explain part of this variation. For all except one participant, home-based cycling was a new discipline, some participants were already physically active regularly while others were not. During the intervention, one participant traveled for 3 weeks, and another was sick for 3 weeks. Such challenges are difficult to avoid in a real-life setting and contribute to the variance in adherence.

The training involved a large volume of moderate- to high-intensity intervals, placing considerable strain on the cardiovascular system. Yet, no adverse events occurred that required medical attention or hindered ongoing participation, confirming that the home-based cycling was safely administered. One participant reported experiencing leg cramps following the most high-intensity workout sessions, while another complained about pressure on his prostate. Although leg cramps are uncomfortable, they are a common condition experienced by recreational and competitive athletes. Most leg cramps, particularly those that occur during exercise or at night, are harmless and typically resolve on their own within a few minutes (19). In addition, prostate discomfort or pain is fairly common among male cyclists, especially those who ride frequently or for long distances (20). Ergonomic bike seats or adjusting riding posture may help. In both cases, no treatment was necessary, and the participants were able to continue with the intervention, although it did affect their motivation for training.

Most participants (73%) prioritized training together with the group at least twice a week, highlighting the value of regular group-based sessions supervised by a coach. However, no correlation was found between training volume and compliance in the group-based sessions implying that group-based training may not be equally important for all individuals. This aligns with a study where 27% of 170 individuals with CP, ranging in age from 4 to 66, showed a preference for solo workout settings (21). The current intervention allowed for individual choices in how to carry out the planned training, thereby accommodating personal preferences for social and supervised training. Addressing personal preferences related to the self-monitoring aspects embedded in the virtual cycling environment is somewhat more challenging. From the evaluation questionnaire, it was obvious that for most participants, the quantitative measures served as a motivating factor, but for one individual, this self-monitoring aspect had the opposite effect. Once again, it emphasizes the importance of aligning exercise treatment with personal preferences to ensure adherence (21).

### Physical measurements

The participants’ cycling performance improved significantly from baseline to post-intervention, as measured by the FTP tests, suggesting that home-based biking can enhance aerobic capacity. This is an important finding as the primary impairments associated with CP include reduced cardiorespiratory fitness and reduced muscle strength. Moreover, exercise approaches that enable individuals with CP to train at moderate to high intensities present a significant challenge (5). Ryan et al. (22) showed that the time participating in moderate physical activity is associated with a reduction in risk factors for cardiometabolic diseases, such as elevated blood pressure and abdominal obesity in adults with CP. The home setup with a stationary bicycle enabled participants to engage in moderate- to high-intensity training by adjusting the resistance to their individual capacity during each workout, ensuring optimized progress while maintaining a comparable level of challenge for all participants.

The challenge was further ensured by a progression in the training program during the intervention. Another home-based study with progressive training among 16 adults with CP (GMFCS I-III) demonstrated an increase in the maximal gait speed after 6 weeks of uphill treadmill training (23). This indicates that adults with CP can effectively conduct progressive aerobic exercise at home, eliminating transportation as a barrier to participating in exercise activities.

Much of the literature published on exercise interventions for adults with CP has focused on the effects of resistance training and strengthening, with limited research exploring the connection between these interventions and aspects of functional activities (18, 24). In this study, we observed improvements in the TUG and STS tests after the intervention, suggesting that increases in cycling performance and aerobic capacity may lead to enhanced functional activity levels. Studies involving strength interventions have demonstrated improvements in the TUG test (25) and the STS test (26) after 10 weeks, however, another study found no improvement in functional mobility after 8 weeks as measured with the TUG test (27). Two studies examining the effects of dance interventions also reported significant improvements in TUG and other balance-related outcomes after 8–12 weeks (28, 29). Nonetheless, given the limited number of studies, small sample sizes ranging from 5 to 10 participants, and high heterogeneity, the effect of different exercise approaches on improving functional activity levels in adults with CP requires further investigation.

### Participant-reported outcomes

While most research has been carried out on the effectiveness of exercise interventions in adults with CP, high-quality evidence is lacking on outcomes that are important to individuals (i.e., quality of life, pain, mood, participation, fatigue, and self-efficacy) (30). In the present study, we found no changes in the subjective perception of fatigue, pain, well-being, sleep, or self-efficacy as measured with validated questionnaires. Unfortunately, only 60% (n = 13) of the current sample completed the questionnaires, limiting the interpretability of the results. Previous studies have observed increased self-efficacy, self-esteem, and mood after group exercise programs (28, 31). Similarly, a qualitative study reports that most of the participants felt that taking part in physical activity interventions had a positive impact on mental well-being and confidence (32). Yet another study involving a small group program of balance training did not observe changes in quality of life or fatigue (33). A lifestyle intervention (comprising physical activity and counseling) found an intervention effect on fatigue measures by CIS-f (Checklist Individual Strength) and on health-related quality of life with respect to bodily pain and mental health. However, no effect was found on fatigue, as measured by FSS, or self-efficacy (34). These conflicting results highlight the need for further research on the impact of exercise on outcomes considered important by adults with CP (18, 35). We did not find any studies that have investigated the impact of exercise on sleep among adults with CP. However, a recent review has concluded that engaging in moderate- to high-intensity exercise has a significant effect on the subjective sleep quality of individuals with Parkinson’s disease (36). While no conclusions can be drawn from participants’ subjective perception of sleep, fatigue, pain, well-being, or self-efficacy in this study, the feedback from the evaluation questionnaires offers optimism for advancing the present intervention to the next stage with more participants and a longer time frame. The long-term aspect is important, as most studies only report outcomes over a short period, and improvements in quality of life and increased participation may require a long time to establish (37).

### Training together while being apart—opportunities and challenges

Recent reviews of exercise interventions for adults with CP emphasize the importance of considering individual needs and preferences, along with the accessibility and sustainability of exercise opportunities (18, 24, 35). With a home-based setup, even high-intensity exercise becomes easily accessible, and the virtual cycling environment can largely be adapted to accommodate individual mobility and abilities. Furthermore, the virtual cycling environment is interactive and has incorporated elements of gamification that may help maintain motivation over time (38). Although much is given from the virtual environment, some motivational factors may be difficult to replace such as exercising together with peers and being supervised by a coach. The online group sessions via Teams may address these challenges by being led by a coach and introducing a mutual obligation among participants, which can promote a sense of belonging (10, 38). Thus, the group session and the virtual environment may facilitate participation and motivation, but the setup may also introduce technical difficulties such as automatic updates and Wi-Fi issues. In the present study, the program Teams was used to support group sessions, introducing another technical system to handle, besides the virtual biking environment Zwift. Thus, while the technical setup can be motivating, it may also pose challenges. However, this field is evolving rapidly, and solutions are constantly improving and being developed to reduce technical issues and meet the needs of the individual.

### Implications

Virtual cycling at home is a safe and feasible approach to increasing moderate- to high-intensity exercise among individuals with activity limitations. The results suggest that a 12-week intervention can enhance physical capacity and improve functional activity outcomes. The coach-led and group-based sessions may support social engagement, facilitate motivation, and ensure proper progression and variety in training. A future study with a longer timeframe and a larger sample size and a control group is needed to confirm these findings and clarify the significance of the various elements involved.

## Data Availability

The raw data supporting the conclusions of this article will be made available by the authors, without undue reservation.
