# Supplementary figures and images for: The feasibility and efficacy of coach-led virtual home-based cycling among individuals with cerebral palsy
Source: Front Neurol. 2025 Jul 15;16:1604061. doi: 10.3389/fneur.2025.1604061 (PMC12306483; doi:10.3389/fneur.2025.1604061)

Appendix 1: Modified Functional Threshold Power (FTP) Test

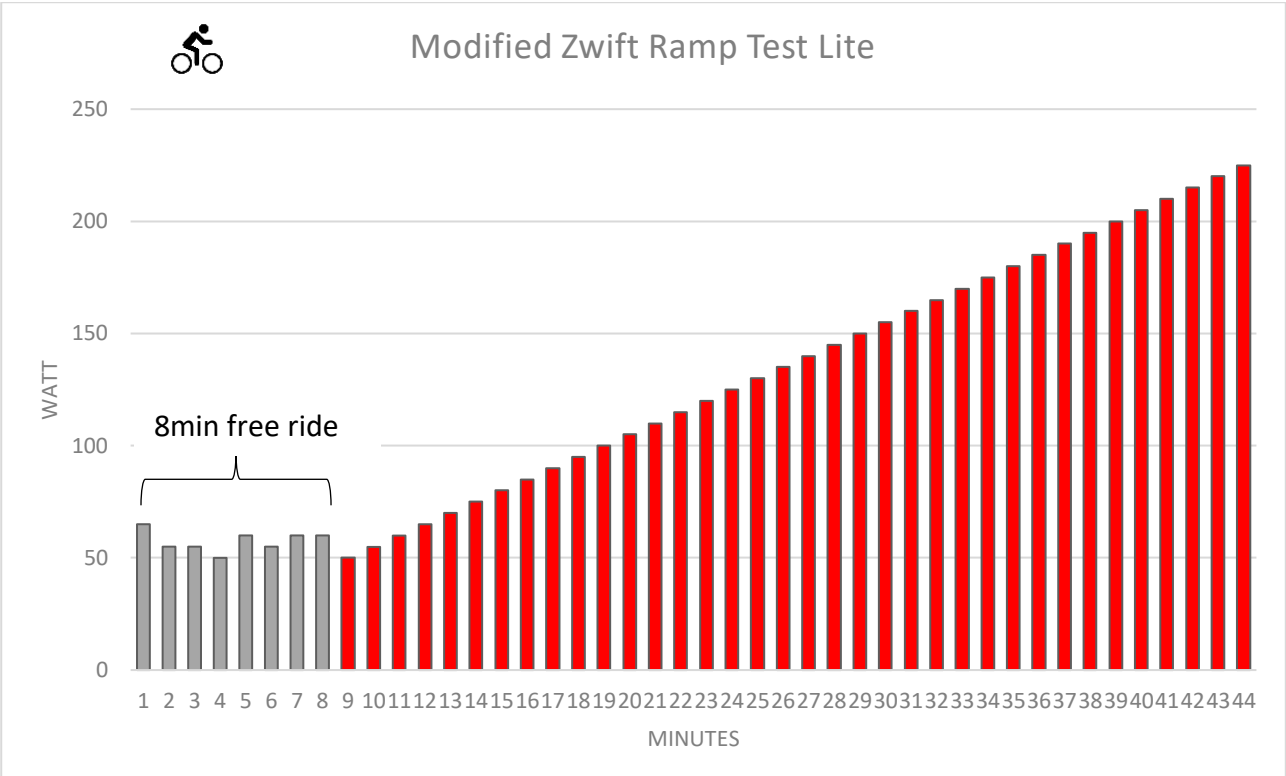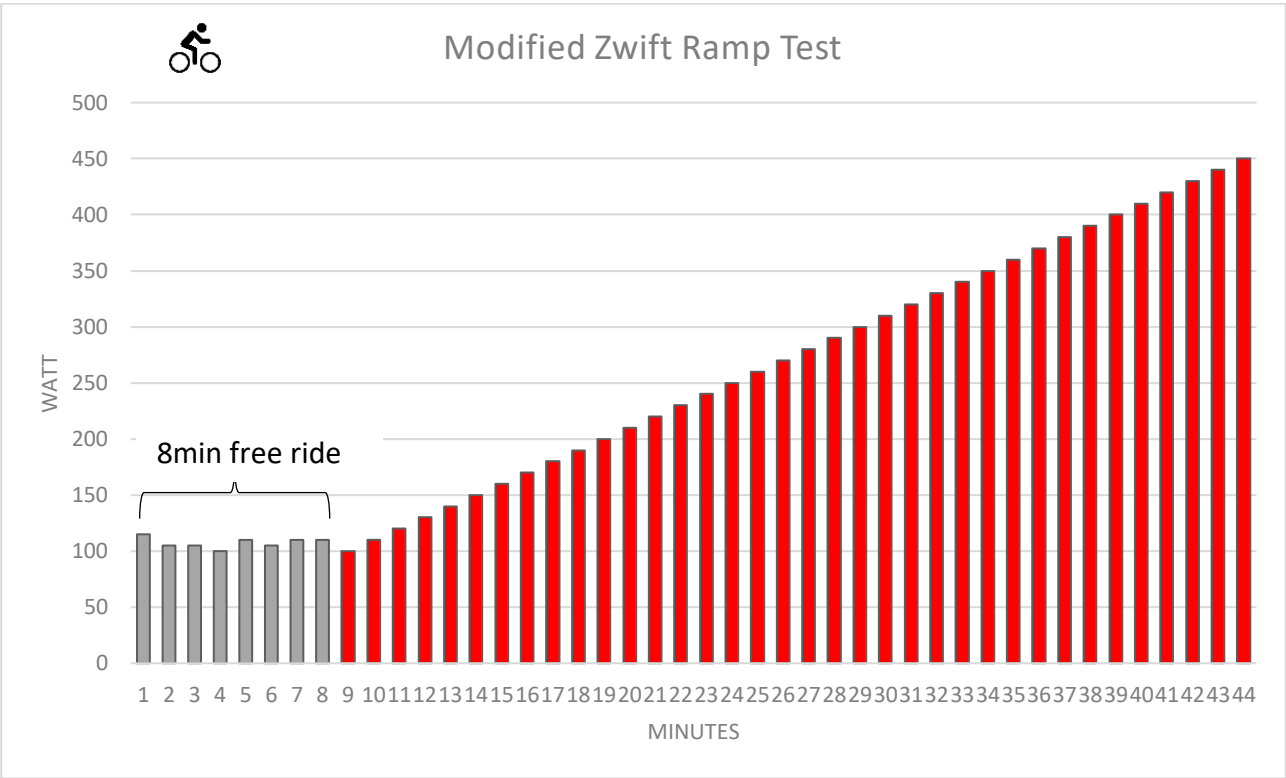

Supplement: Supplementary file 1 [file Data_Sheet_1.pdf]
