# Supplementary material for: The feasibility and efficacy of coach-led virtual home-based cycling among individuals with cerebral palsy
Source: Front Neurol. 2025 Jul 15;16:1604061. doi: 10.3389/fneur.2025.1604061 (PMC12306483; doi:10.3389/fneur.2025.1604061)

Appendix 2: Training Schedule & Load

**Week**	**Day (Session)**	**Training time**	**Teams online**	**Zwift Workout**	**Category**	**Minutes**
1	Monday (1)	18.00	17.50	Fit og Fun	30 minutes to burn	21
	Tuesday (2)	18.00	17.50	Alpha	30 minutes to burn	30
	Thursday (3)	18.00	17.50	Weekend Tune Up	30 minutes to burn	27
2	Monday (4)	18.00	17.50	Flash Burn	30 minutes to burn	27
	Tuesday (5)	18.00	17.50	Under Over	30 minutes to burn	27
	Thursday (6)	18.00	17.50	Sevens	30 minutes to burn	30
3	Monday (7)	18.00	17.50	Expand	30 minutes to burn	30
	Tuesday (8)	18.00	17.50	Emilys Short Mix	30 minutes to burn	30
	Thursday (9)	18.00	17.50	Devedeset Lite	30-60 min to burn	32
4	Monday (10)	18.00	17.50	Challenge Alpe de Zwift	Route	120
	Tuesday (11)	18.00	17.50	Foundation	30-60 min to burn	48
	Thursday (12)	18.00	17.50	Ove, Under and Beyond	30-60 min to burn	46
5	Monday (13)	18.00	17.50	Hairpin Wizard	30-60 min to burn	44
	Tuesday (14)	18.00	17.50	Short Sweet Spot Tune Up	30-60 min to burn	45
	Thursday (15)	18.00	17.50	The Wringer	30-60 min to burn	43
6	Monday (16)	18.00	17.50	Pyramid Cadence Efforts	30-60 min to burn	56
	Tuesday (17)	18.00	17.50	Step by Step	30-60 min to burn	40
	Thursday (18)	18.00	17.50	Through the Zones #1	30-60 min to burn	55
7	Monday (19)	18.00	17.50	At/Over/Under	Threshold	47
	Tuesday (20)	18.00	17.50	Jon's Mix	30-60 min to burn	54
	Thursday (21)	18.00	17.50	Up and Down	30-60 min to burn	35
8	Monday (22)	18.00	17.50	Challenge Timetrial	Route	43
	Tuesday (23)	18.00	17.50	Endurance #1	Endurance	60
	Thursday (24)	18.00	17.50	EPOC	Sprinting	52
9	Monday (25)	18.00	17.50	Cadence Bursts	Endurance	63
	Tuesday (26)	18.00	17.50	Exemplar	Threshold	60
	Thursday (27)	18.00	17.50	Workout 3	Endurance	60
10	Monday (28)	18.00	17.50	Gearing	Sprinting	67

	Tuesday (29)	18.00	17.50	Endurance #2	Endurance	62
	Thursday (30)	18.00	17.50	Cruise intervals #1	Threshold	54
11	Monday (31)	18.00	17.50	Twelve Spikes	Endurance	60
	Tuesday (32)	18.00	17.50	EF Pro Cyclings yellow day workout	Sprinting	55
	Thursday (33)	18.00	17.50	Pacing #1	Endurance	58
12	Monday (34)	18.00	17.50	Challenge Ventop	Route	120
	Tuesday (35)	18.00	17.50	Endurance #3	Endurance	66
	Thursday (36)	18.00	17.50	Cruise intervals #4	Threshold	59

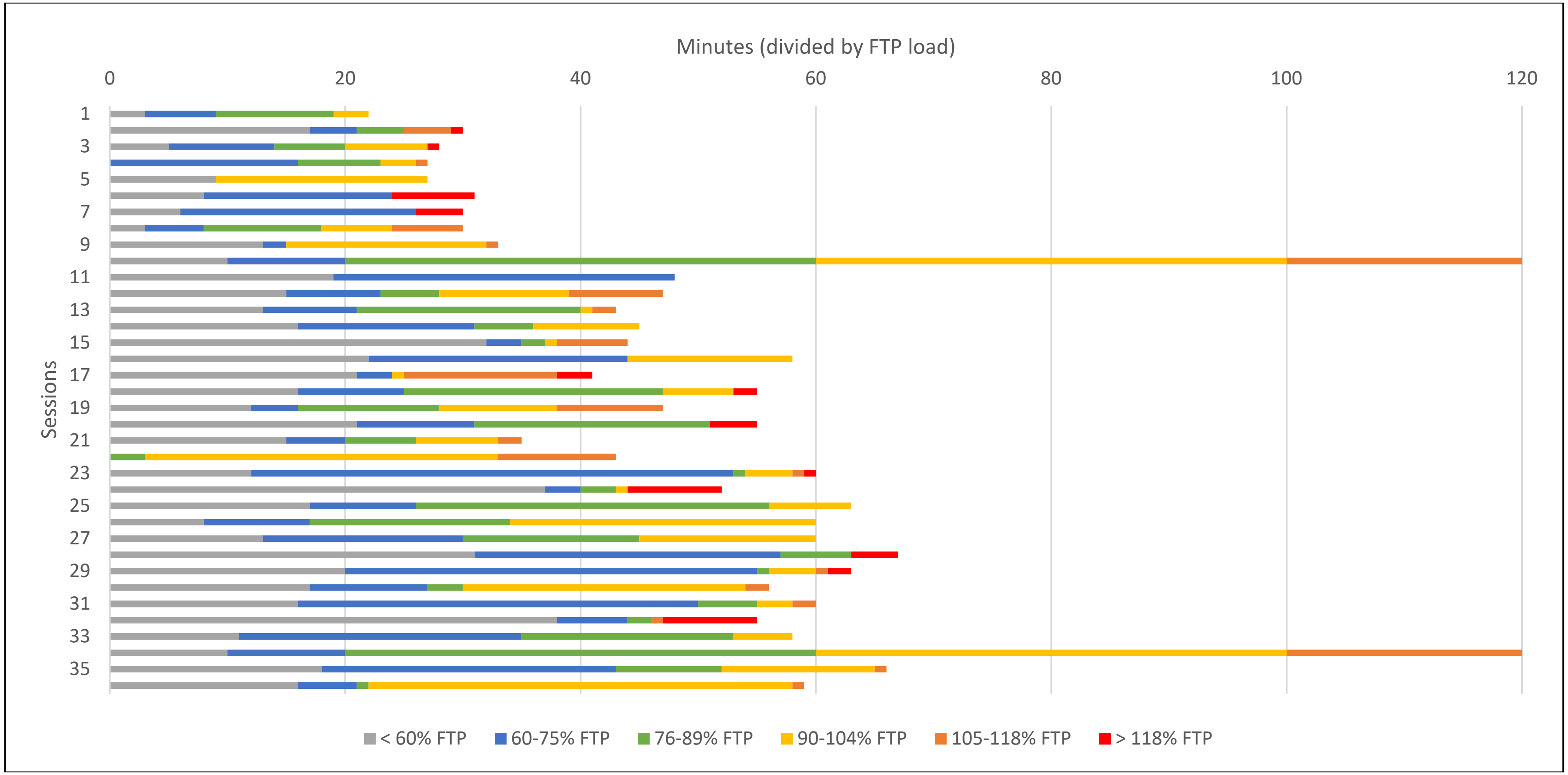

Supplement: Supplementary file 2 [file Data_Sheet_2.pdf]
